# Thanks to Reviewers 2024

**DOI:** 10.5334/pb.1402

**Published:** 2025-05-27

**Authors:** Editorial Team

**Affiliations:** 1 Psychologica Belgica, Belgium

## Abstract

All manuscripts published in Psychologica Belgica have been assessed conscientiously and unselfishly by expert reviewers. The quality of our journal totally depends on their valuable and constructive criticisms to the authors. Both the editors and the authors highly appreciate the input and dedication of all our reviewers. Many thanks.

Ellen Greimel

Marcos Romero

Pascalle Spaan

Elinor Waite

Nathalie Delobbe

Dirk van Dierendonck

ntsame-sima Murielle

Andrea Chirico

Francisca Rodriguez

Veronique Maheux-Caron

Etienne Quertemont

Pierre De Oliveira

Pierre Le Denmat

Marc Macé

Patricia Bijttebier

Sofie Weyn

Wan-Lan Chen

Berre Deltomme

Hayley Pearce

Tim Vestner

Asanka Gunasekara

Elodie Pools

Bruno Rossion

Joachim Waterschoot

Lorenz Rapp

Wolf Mehling

Li Haoran

Leentje Vervoort

Liesbeth Bogaert

Annelies Raes

Angelika Stefan

Man Chen

Lien Goossens

Patrick Onghena

Kimberly Barchard

Thom Volker

Iuliana Mihaela Lazar

Vera Hoorens

Oscar Astivia

Marie-Anne Schelstraete

Christian Blötner

David Wo

Andreas Stengel

Thorsten Rudroff

Jan Van Der Linden

Sandra Invernizzi

Celine Baeyens

Gerly Tamm

